# Supplementary material for: Exploring Cyberaggression and Mental Health Consequences among Adults: An Italian Nationwide Cross-Sectional Study
Source: Int J Environ Res Public Health. 2023 Feb 12;20(4):3224. doi: 10.3390/ijerph20043224 (PMC9958796; doi:10.3390/ijerph20043224)
Supplement: Supplementary file 1 [file ijerph-20-03224-s001.zip › ijerph-2195223-supplementary.pdf]

Supplementary

Supplementary Table S1. Description of online aggressive behaviours from victims', authors' and bystanders' point of view and platforms where they frequently occur.

Online behaviours	Overall sample<br>N (%)<br>N= 446
**Victim of**	
Harassment	90 (20.2)
Defamation	49 (11.0)
Non-consensual sharing of intimate content	15 (3.4)
Stalking	46 (10.3)
Discrimination	47 (10.6)
Insult	122 (27.4)
ID Thief	36 (8.1)
Never occurred	239 (53.7)
Occurred over the last two weeks	26 (12.7)
**Author of**	
Harassment	7 (1.6)
Defamation	4 (0.9)
Non-consensual sharing of intimate content	6 (1.4)
Stalking	0 (0)
Discrimination	5 (1.1)
Insult	49 (11.1)
ID Thief	3 (0.7)
Never occurred	383 (85.9)
Occurred over the last two weeks	9 (15.5)
**Bystander of**	
Harassment	113 (25.3)
Defamation	216 (48.4)
Non-consensual sharing of intimate content	77 (17.3)
Stalking	74 (16.6)
Discrimination	285 (63.9)
Insult	285 (63.9)
ID Thief	139 (31.2)
Never occurred	102 (22.9)
Occurred over the last two weeks	161 (47.5)
**Platforms of occurrence**	
**Social Network**	335 (96.0)
Facebook	240 (71.6)
Instagram	80 (23.9)
Twitter	21 (6.3)
Tik-Tok	5 (1.5)
**Messaging Apps**	90 (25.8)
WhatsApp	41 (45.6)
Messenger	8 (8.9)
Telegram	22 (24.4)
**Forums, Reddit, Blogs**	23 (6.6)

Forum	2 (8.7)
Magazine	5 (21.7)
Reddit	5 (21.7)
**Video Platforms**	45 (12.9)
YouTube	34 (75.6)
**Dating Apps**	15 (4.3)
Tinder	5 (33.3)
Grindr	8 (53.3)
**Means of CyA communication**	
Voice message	16 (4.6)
Text message	100 (28.7)
Comment	289 (83.0)
Post	234 (67.2)
Picture	131 (37.6)
Meme	78 (22.4)
Video	64 (18.4)
E-mail	6 (1.7)
**Changes during the pandemic**	
Increased	186 (41.7)
Decreased	6 (1.3)
Unchanged	75 (16.8)
No idea	179 (40.1)
**Topics**	
Politic	242 (69.9)
Science	116 (33.5)
Ethnicity	226 (65.3)
Female gender	183 (52.9)
Sexual orientation	223 (64.5)
Gender identity	161 (46.5)
Disability	78 (22.5)
Religion	75 (21.7)
Body looking	167 (48.3)
Public figure	124 (35.8)

Abbreviations **CyA** cyber aggression

Supplementary Table S2. Context of online aggression and comparison with face-to-face violence

Variable	Overall sample	Being Author of CyA			Being Victim of CyA		
		No	Yes	p-value	No	Yes	p-value
	N (%)	N (%)	N (%)		N (%)	N (%)	
	N= 446	383 (85.9)	60 (13.5)		239 (53.7)	206 (46.2)	
**Victim of:**							
**Harassment**							
No	355 (79.8)	315 (89.2)	38 (10.8)	**0.001**	-	-	-
Yes	90 (20.2)	67 (75.3)	22 (24.7)		-	-	
**Defamation**							
No	396 (89.0)	348 (88.1)	47 (11.9)	**0.003**	-	-	-
Yes	49 (12.0)	34 (72.3)	13 (27.7)		-	-	
**Non-consensual sharing of intimate content**							
No	430 (96.6)	368 (86.2)	59 (13.8)	0.427	-	-	-
Yes	15 (3.4)	14 (93.3)	1 (6.7)		-	-	
**Stalking**							
No	399 (89.7)	351 (88.4)	46(11.6)	**< 0.001**	-	-	-
Yes	46 (10.3)	31 (68.9)	14(31.1)		-	-	
**Discrimination**							
No	398 (89.4)	352 (89.1)	43 (10.9)	**< 0.001**	-	-	-
Yes	47 (10.6)	30 (63.8)	17 (36.2)		-	-	
**Insult**							
No	323 (72.6)	309 (95.7)	14 (4.3)	**< 0.001**	-	-	-
Yes	122 (27.4)	73 (61.3)	46(38.7)		-	-	
**ID Thief**							
No	409 (91.9)	354 (87.2)	52 (12.8)	0.114	-	-	-
Yes	36 (8.1)	28 (77.8)	8 (22.2)		-	-	
**Never occurred**							
No	206 (46.3)	149 (73.4)	54 (26.6)	**< 0.001**	-	-	-
Yes	239 (53.7)	233 (97.5)	6 (2.5)		-	-	
**Occurred over the last two weeks**							
No	178 (87.3)	132 (75.4)	43 (24.6)	0.057	-	-	-
Yes	26 (12.7)	15 (57.7)	11 (42.3)		-	-	
**Author of:**							
**Harassment**							
No	436 (98.4)	-	-	-	238 (54.7)	197 (45.3)	**0.033**
Yes	7 (1.6)	-	-		1 (14.3)	6 (85.7)	
**Defamation**							
No	439 (99.1)	-	-	-	238 (54.3)	200 (45.7)	0.241
Yes	4 (0.9)	-	-		1 (25.0)	3 (75.0)	
**Non-consensual sharing of intimate content**							
No	437 (98.6)	-	-	-	239 (54.8)	197 (45.2)	**0.007**
Yes	1 (1.4)	-	-		0 (0.0)	6 (100.0)	
**Stalking**							
No	443 (100)	-	-	-	239 (54.1)	203 (45.9)	-
Yes	0 (0.0)	-	-		0 (0.0)	0 (0.0)	
**Discrimination**							
No	438 (98.9)	-	-	-	239 (54.7)	198 (45.3)	**0.015**
Yes	5 (1.1)	-	-		0 (0.0)	5 (100.0)	

**Insult**							
No	394 (88.9)	-	-	-	235 (59.8)	158 (40.2)	**< 0.001**
Yes	49 (11.1)	-	-		4 (8.2)	45 (91.8)	
**ID Thief**							
No	440 (99.3)	-	-	-	238 (54.2)	201 (45.8)	0.470
Yes	3 (0.7)	-	-		1 (33.3)	2 (66.7)	
**Never occurred**							
No	60 (13.5)	-	-	-	6 (10.0)	54 (90.0)	**< 0.001**
Yes	383 (86.5)	-	-		233 (61.0)	149 (39.0)	
**Occurred over the last two weeks**							
No	49 (84.5)	-	-	-	5 (10.2)	44 (89.8)	0.935
Yes	9 (15.5)	-	-		1 (11.1)	8 (88.9)	
**Ftf-V Victims**							
Never	207 (46.9)	191 (92.3)	16 (7.7)	**0.006**	138(66.7)	69 (33.3)	**< 0.001**
Only childhood	184 (41.7)	154 (84.2)	29(15.8)		88 (48.1)	95 (51.9)	
Only Adulthood	19 (4.3)	15 (78.9)	4 (21.1)		8 (42.1)	11 (57.9)	
Both	31 (7.0)	22 (73.3)	8 (26.7)		4 (12.9)	27 (87.1)	
**Ftf-V Authors**							
Never	385 (87.1)	349(90.9)	35 (9.1)	**< 0.001**	220 (57.3)	164 (42.7)	**0.002**
Only childhood	49 (11.1)	29 (60.4)	19(39.6)		17 (34.7)	32 (65.3)	
Only Adulthood	4 (0.9)	3 (75.0)	1 (25.0)		1 (25.0)	3 (75.0)	
Both	4 (0.9)	1 (25.0)	3 (75.0)		0 (0.0)	4 (100.0)	
**Ftf-V Bystanders**							
Never	141 (32.0)	133 (94.3)	8 (5.7)	**0.001**	96 (68.6)	44 (31.4)	**< 0.001**
Only childhood	189 (43.0)	160 (85.1)	28 (14.9)		100 (52.9)	89 (47.1)	
Only Adulthood	49 (11.1)	43 (87.8)	6 (12.2)		25 (51.0)	24 (49.0)	
Both	61 (13.9)	44 (73.3)	16 (26.7)		17 (27.9)	44 (72.1)	
**Context in which generally CyA occurs according to respondents' opinion**							
Online	230 (52.0)	195 (85.2)	34 (14.8)	0.284	118 (51.3)	112 (48.7)	0.459
In person	81 (18.3)	69 (85.2)	12 (14.8)		47 (58.8)	33 (41.3)	
No idea	131 (29.6)	118 (90.2)	12 (9.8)		73 (55.7)	58 (44.3)	
**Perception of online anonymity**							
Not at all-low	255 (79.6)	310 (87.3)	43 (12.1)	**0.002**	182 (51.3)	172 (48.4)	**0.029**
Medium - high	62 (13.9)	45 (68.2)	17 (25.7)		34 (51.5)	28 (42.4)	
Irrelevant	29 (6.5)	28 (96.5)	0 (0.0)		23 (79.3)	6 (20.7)	
**Perception of online disinhibition**							
Not at all-low	311 (69.8)	281 (90.3)	27 (8.7)	**<0.001**	182 (58.5)	128 (41.1)	**0.001**
Medium - high	96 (21.6)	66 (68.7)	30 (31.3)		34 (35.4)	62 (64.6)	
Irrelevant	39 (8.7)	36 (92.3)	3 (7.7)		23 (59.0)	16 (41.0)	

P-value obtained via chi-squared test.
Overall sample: column percentage
Being Author/Victim of CyA: row percentage
Abbreviations **Ftf V** face to face violence; **CyA** cyber aggression; **ID** identity

Supplementary Table S3. Mental health and psychosomatic symptoms for authors and victims of cyber aggression.

Variable	Overall sample	Being Author of CyA			Being Victim of CyA		
		No	Yes	p-value	No	Yes	p-value
	N (%)	N (%)	N (%)		N (%)	N (%)	
	N= 446	383 (85.9)	60 (13.5)		239 (53.7)	206 (46.2)	
**Fatigue**							
Rarely-never	242 (72.3)	207 (85.9)	34 (14.1)	0.055	120 (49.8)	121 (50.2)	**< 0.001**
Very often-often	93 (27.7)	71 (77.2)	21 (22.8)		20 (21.5)	73 (78.5)	
**Anhedonia**							
Rarely-never	217 (64.8)	184 (85.2)	32 (14.8)	0.256	108 (50.0)	108 (50.0)	**< 0.001**
Very often-often	118 (35.2)	94 (80.3)	23 (19.7)		32 (27.1%)	86 (72.9)	
**Sadness**							
Rarely-never	141 (32.0)	105(79.5)	27(20.5)	0.117	56 (42.4)	76 (57.6)	0.879
Very often-often	189 (43.0)	173(86.1)	28 (13.9)		84 (41.6)	118 (58.4)	
**Anxiety**							
Rarely-never	170 (50.8)	141(83.4)	28 (16.6)	0.979	88 (52.1)	81 (47.9)	**< 0.001**
Very often-often	165 (48.2)	137(83.5)	27 (16.5)		52 (31.5)	113 (68.5)	
**Rumination**							
Rarely-never	195 (58.2)	169(87.1)	25 (12.9)	0.035	70 (53.8)	60 (46.2)	**< 0.001**
Very often-often	140 (41.8)	109(78.4)	30 (21.6)		70 (34.3)	134 (65.7)	
**Anger**							
Rarely-never	130 (38.8)	116 (89.2)	14 (10.8)	**0.024**	70 (53.8)	60 (46.2)	**< 0.001**
Very often-often	205 (61.1)	162 (79.8)	41(20.2)		70 (34.3)	134 (65.7)	
**Fear**							
Rarely-never	243 (72.5)	198 (81.8)	44 (18.2)	0.182	109 (45.0)	133 (55.0)	0.060
Very often-often	92 (27.5)	80 (87.9)	11(12.1)		31 (33.7)	61 (66.3)	
**Eating alterations**							
Rarely-never	283 (84.5)	237 (84.0)	45 (16.0)	0.518	123 (43.6)	159 (56.4)	0.142
Very often-often	52 (15.6)	41 (80.4)	10 (19.6)		17 (32.7)	35 (67.3)	
**Shame**							
Rarely-never	233 (69.6)	237 (84.0)	45 (16.0)	0.518	123 (43.6)	159 (56.4)	0.142
Very often-often	102 (30.4)	41 (80.4)	10 (19.6)		17 (32.7)	35 (67.3)	
**Suicidal Thoughts/attempts**							
Rarely-never	318 (95.0)	264 (83.5)	52 (16.5)	0.897	137 (43.2)	180 (56.8)	**0.037**
Very often-often	17 (5.1)	14 (82.4)	3 (17.6)		3 (17.6)	14 (82.4)	
**Revenge**							
Rarely-never	279 (83.3)	230 (83.0)	47 (17.0)	0.622	121 (43.4)	158 (56.6)	0.225
Very often-often	56 (16.7)	48 (85.7)	8 (14.3)		19 (34.5)	36 (65.5)	
**Sense of belonging to a group**							
Rarely-never	300 (89.5)	254 (85.2)	44 (14.8)	**0.012**	128 (42.8)	171 (57.2)	0.334
Often-very often	35 (10.5)	24 (68.6)	11 (31.4)		12 (34.3)	23 (65.7)	
**Sense of pride or justice**							
Rarely-never	251 (74.9)	207 (83.1)	42 (16.9)	0.767	110 (43.8)	141 (56.2)	0.219
Very often-often	84 (25.1)	71 (84.5)	13 (15.5)		30 (36.1)	53 (63.9)	
**Muscle Stiffness**							
Rarely-never	281 (83.9)	237 (84.9)	42 (15.1)	0.102	127 (45.4)	153 (54.6)	**0.004**
Very often-often	54 (16.2)	41 (75.9)	13 (24.1)		13 (24.1)	41 (75.9)	
**Headache**							
Rarely-never	290 (86.6)	246 (85.1)	43 (14.9)	**0.039**	134 (46.4)	155 (53.6)	**< 0.001**
Very often-often	45 (13.5)	32 (72.7)	12(27.3)		6 (13.3)	39 (86.7)	

**Stomacache**							
Rarely-never	271 (80.9)	230 (85.2)	40 (14.8)	0.083	129 (47.8)	141 (52.2)	**< 0.001**
Very often-often	64 (19.1)	48 (76.2)	15 (23.8)		11 (17.2)	53 (82.8)	
**Gastro-intestinal symptoms**							
Rarely-never	288 (86.0)	242 (84.3)	45 (15.7)	0.304	133 (46.3)	154 (53.7)	**< 0.001**
Very often-often	47 (14.0)	36 (78.3)	10 (21.7)		7 (14.9)	40 (85.1)	
**Immunodeficiency signs**							
Rarely-never	313 (93.5)	262 (84.2)	49 (15.8)	0.160	138 (44.2)	174 (55.8)	**0.001**
Very often-often	22 (6.6)	16 (72.7)	6 (27.3)		2 (9.1)	20 (90.9)	
**Sleep alterations**							
Rarely-never	246 (73.4)	207 (84.5)	38 (15.5)	0.409	121 (49.4)	124 (50.6)	**< 0.001**
Very often-often	89 (26.6)	71 (80.7)	17(19.3)		19 (21.3)	70 (78.7)	

P-value obtained via chi-squared test.
Overall sample: column percentage
Being Author/Victim of CyA: row percentage
Abbreviation **CyA** cyber aggression

Supplementary Table S4. Consequent troubles for victims and authors of cyber aggression.

Variable	Overall sample	Being Author of CyA			Being Victim of CyA		
		No	Yes	p-value	No	Yes	p-value
	N (%)	N (%)	N (%)		N (%)	N (%)	
	N= 446	383 (85.9)	60 (13.5)		239 (53.7)	206 (46.2)	
**Reputation damage**							
Rarely-never	310 (94.5)	259 (84.1)	49 (15.9)	0.188	136 (44.0)	173 (56.0)	**< 0.001**
Very often-often	18 (5.5)	13 (72.2)	5 (27.8)		0 (0.0)	18 (100.0)	
**Workplace and/or university issues**							
Rarely-never	315 (96.0)	263 (84.0)	50 (16.0)	0.160	135 (43.0)	179 (57.0)	**0.011**
Very often-often	13 (4.0)	9 (69.2)	4 (30.8)		1 (7.7)	12 (92.3)	
**Relations conflicts**							
Rarely-never	295 (90.0)	255 (87.0)	38 (13.0)	**< 0.001**	132 (44.9)	162 (55.1)	**< 0.001**
Very often-often	33 (10.0)	17 (51.5)	16 (48.5)		4 (12.1)	29 (87.9)	
**Reduced SN Usage. SN account cancellation**							
Rarely-never	283 (86.3)	239 (85.1)	42 (14.9)	0.050	126 (44.7)	156 (55.3)	0.005
Very often-often	45 (13.7)	33 (73.3)	12 (26.7)		10 (22.2)	35 (77.8)	
**Life changes (relocation. job change)**							
Rarely-never	313 (95.5)	259 (83.3)	52 (16.7)	0.730	136 (43.6)	176 (56.4)	**0.001**
Very often-often	15 (4.5)	13 (86.7)	2 (13.3)		0 (0.0)	15 (100.0)	
**Legal Actions**							
Rarely-never	325 (99.1)	269 (83.3)	54 (16.7)	0.438	136 (42.0)	188 (58.0)	0.142
Very often-often	3 (0.9)	3 (100.0)	0 (0.0)		0 (0.0)	3 (100.0)	
**Postal police charge**							
No	315 (96.0)	262 (83.4)	52 (16.6)	0.992	135 (43.0)	179 (57.0)	**0.011**
Yes	13 (4.0)	10 (83.3)	2 (16.7)		1 (7.7)	12 (92.3)	

P-value obtained via chi-squared test.
Overall sample: column percentage
Being Author/Victim of CyA: row percentage
Abbreviation **CyA** cyber aggression; **SN** social network

Supplementary Table S5. Secondary Outcomes: Risk of anxiety and depressive disorders (positive GAD-2 and PHQ-2 score) and socio-demographic characteristics.

**Characteristic**	**Overall sample**	**Risk of anxiety (GAD-2)**			**Risk of depression (PHQ-2)**		
		**No**	**Yes**	**p-value**	**No**	**Yes**	**p-value**
	**N (%)**	**N (%)**	**N (%)**		**N (%)**	**N (%)**	
	N= 446	283 (66.0)	146 (34.0)		333(77.6)	96 (22.4)	
**Age**		**Median** 34 **IQR** (26-45.75)	**Median** 28 **IQR** (25-39.75)		**Median** 34 **IQR** (26-45)	**Median** 28 **IQR** (25-36)	
**Gender**							
Men	167 (37.4)	118 (73.3)	43 (26.7)	**0.048**	125 (77.6)	36 (22.4)	0.920
Women	275 (61.7)	163 (61.7)	101 (38.3)		205 (77.7)	59 (22.3)	
Female-to-Male	1 (0.2)	1 (100.0)	0 (0.0)		1 (100.0)	0 (0.0)	
Queer	3 (0.7)	1 (33.3)	2 (66.7)		2 (66.7)	1 (33.3)	
**Sexual Orientation**							
CIS - Heterosexual	390 (87.4)	253 (67.5)	122 (32.5)	0.084	296 (78.9)	79(21.1)	0.086
LGBTQA+pansexual	56 (12.6)	30 (55.6)	24 (44.4)		37 (68.5)	17(31.5)	
**Education**							
Secondary or High School	248 (55.6)	171(71.3)	69 (28.8)	**0.009**	201 (83.8)	39 (16.3)	**0.001**
College or Master/PhD	198 (44.4)	112 (59.3)	77 (40.7)		132(69.8)	57 (30.2)	
**Employment**							
Employed	254 (57.0)	174 (71.0)	71 (29.0)	**0.001**	198 (80.8)	47 (19.2)	**0.002**
Student	145 (32.5)	75 (53.6)	65 (46.4)		95 (67.9)	45 (32.1)	
Retired, housewife, unemployed	43 (9.6)	30 (75.0)	10 (25.0)		36 (90.0)	4 (10.0)	
**Own + parents birthplace**							
Italy	425 (95.3)	270 (66.2)	138(33.8)	0.687	315(77.2)	93 (22.8)	0.362
Born abroad or at least one parent born abroad	21 (4.7)	13 (61.9)	8 (38.1)		18 (85.7)	3 (14.3)	
**Family relationship**							
Good-very good	331 (74.2)	222(69.6)	97 (30.4)	**0.007**	256 (80.3)	63 (19.7)	**0.026**
Bad-very bad	115 (25.8)	61 (55.5)	49 (44.5)		77 (70.0)	33 (30.0)	
**Social life**							
Many people	126 (28.3)	86 (72.9)	32 (27.1)	**0.002**	102 (86.4)	16 (13.6)	**< 0.001**
Some people	293 (65.7)	187 (65.8)	97 (34.2)		219 (77.1)	65 (22.9)	
No contact	16 (3.6)	4 (25.0)	12 (75.0)		7 (43.8)	9 (56.3)	
Social Isolation	11 (2.5)	6 (54.5)	5 (45.5)		5 (45.5)	6 (54.5)	
**Economic status**							
High-Medium	400 (89.7)	257(66.6)	129(33.4)	0.422	308 (79.8)	78 (20.2)	**0.001**
Low-Very low	46 (26.5)	26 (60.5)	17 (39.5)		25 (58.1)	18 (41.9)	
**CAGE-AID**							
Negative	328 (73.5)	222 (69.6)	97 (30.4)	**0.007**	258 (80.9)	61 (19.1)	**0.006**
Positive	118 (26.5)	61 (55.5)	49 (44.5)		75 (68.2)	35 (31.8)	
**Social Network Frequency Use**							
Very often - often	324 (72.6)	208 (66.9)	103(33.1)	0.491	241(77.5)	70 (22.5)	0.121

Rarely - never	122 (37.4)	75 (63.5)	43 (36.5)		92 (78.0)	26 (22.0)	
**Messaging Apps Frequency Use**							
Very often -often	413 (92.6)	163 (41.2)	133(58.8)	0.085	310 (78.3)	86 (21.7)	0.154
Rarely-Never	33 (7.4)	20 (60.6)	13 (39.4)		23 (69.7)	10 (30.3)	
**Video Platforms Frequency Use**							
Very often-often	178 (39.9)	118 (69.0)	53 (31.0)	0.683	130 (76.0)	41 (34.0)	0.322
Rarely-Never	268 (60.1)	165 (63.9)	93 (36.1)		203 (78.7)	55 (21.3)	
**Forums - Blogs Frequency Use**							
Very often-often	111 (24.9)	67 (63.2)	39 (54.8)	0.831	79 (74.5)	27 (25.5)	0.271
Rarely-Never	335 (75.1)	216 (66.9)	107(33.1)		254 (78.6)	69 (21.4)	
**Dating Apps Frequency Use**							
Very often-often	34 (7.6)	26 (76.5)	8 (23.5)	0.264	27 (79.4)	7 (20.6)	0.851
Rarely-Never	412 (92.4)	257 (65.1)	138(34.9)		306 (77.5)	89 (23.5)	

P-value obtained via chi-squared test.
Overall sample: column percentage
Risk of anxiety (GAD-2)/ Risk of depression (PHQ-2): row percentage
Abbreviation **IQR** interquartile range; **LGBTQA** lesbian, gay, bisexual, transgender, queer/questioning, intersex, and allies; **GAD-2** Generalized Anxiety Disorder 2-item; **PHQ-2** Patient Health Questionnaire 2-item; **CAGE-AID**: Cut down. Annoyed. Guilty. Eye opener Adapted to Include Drugs

Supplementary Table S6. Risk of anxiety and depressive symptoms for victims. perpetrators and bystanders

Characteristic	Overall sample	Risk of anxiety (GAD-2)			Risk of depression (PHQ-2)		
		No	Yes	p-value	No	Yes	p-value
	N (%)	N (%)	N (%)		N (%)	N (%)	
	N=446	283 (66.0)	146 (34.0)		333 (77.6)	96 (22.4)	
<u>Victim of</u>							
**Harassment**							
No	355 (79.8)	235 (68.5)	108(31.5)	**0.036**	267 (77.8)	76 (22.2)	0.786
Yes	90 (20.2)	48 (56.5)	37 (43.5)		65 (76.5)	20 (23.5)	
**Defamation**							
No	396 (89.0)	259 (68.0)	122(32.0)	**0.021**	381 (89.0)	298 (78.2)	0.362
Yes	49 (12.0)	24 (51.1)	23 (48.9)		47 (11.0)	34 (72.3)	
**Non-consensual sharing of intimate content**							
No	430 (96.6)	278 (67.3)	135(32.7)	**0.006**	322 (78.0)	91 (22.0)	0.303
Yes	15 (3.4)	5 (33.3)	10 (66.7)		10 (66.7)	5 (33.3)	
**Stalking**							
No	399 (89.7)	259 (67.1)	127(32.9)	0.195	303 (78.5)	83 (21.5)	0.163
Yes	46 (10.3)	24 (57.1)	18 (42.9)		29 (69.0)	13 (31.0)	
**Discrimination**							
No	398 (89.4)	262 (68.3)	121(31.7)	**0.006**	302 (79.1)	80 (20.9)	**0.034**
Yes	47 (10.6)	22 (47.8)	24 (52.2)		30 (65.2)	16 (34.8)	
**Insult**							
No	323 (72.6)	217 (69.6)	95 (30.4)	**0.014**	249 (79.8)	63 (20.2)	0.069
Yes	122 (27.4)	66 (56.9)	50 (43.1)		83 (71.6)	33 (28.4)	
**ID Thief**							
No	409 (91.9)	257 (65.1)	138(34.9)	0.110	306 (77.5)	89 (22.5)	0.861
Yes	36 (8.1)	26 (78.8)	7 (21.2)		26 (78.8)	7 (21.2)	
**Never**							
No	206 (46.3)	118 (60.5)	77 (39.5)	**0.025**	148 (75.9)	47 (24.1)	0.448
Yes	239 (53.7)	165 (70.8)	68 (29.2)		184 (79.0)	49 (21.0)	
**Over the last 2 weeks**							
No	178 (87.3)	104 (61.5)	65 (38.5)	0.363	131 (77.5)	38 (22.5)	0.296
Yes	26 (12.7)	13 (52.0)	12 (48.0)		17 (68.0)	8 (32.0)	
<u>Author of</u>							
**Harassment**							
No	436 (98.4)	277 (66.0)	143(34.0)	0.762	325 (77.4)	95 (22.6)	0.600
Yes	7 (1.6)	5 (71.4)	2 (28.6)		6 (85.7)	1 (14.3)	
**Defamation**							
No	439 (99.1)	279 (66.0)	144(34.0)	0.704	328 (77.5)	95 (22.5)	0.904
Yes	4 (0.9)	3 (75.0)	1 (25.0)		3 (75.0)	1 (25.0)	
**Non-consensual sharing of intimate content**							
No	437 (98.6)	280 (66.5)	141(35.5)	0.088	328 (77.9)	93 (22.1)	0.104
Yes	1 (1.4)	2 (33.3)	4 (66.7)		3 (50.0)	3 (50.0)	
**Stalking**							
No	443 (100)	282 (66.0)	145(34.0)	-	331 (77.5)	96 (22.5)	-
Yes	0 (0.0)	0 (0.0)	0 (0.0)		0 (0.0)	0 (0.0)	
**Discrimination**							
No	438 (98.9)	279 (66.1)	143(33.9)	0.774	328 (77.7)	94 (22.3)	0.345
Yes	5 (1.1)	3 (60.0)	2 (40.0)		3 (60.0)	2 (40.0)	

**Insult**							
No	394 (88.9)	256 (67.0)	126(33.0)	0.216	301 (78.8)	81 (21.2)	0.065
Yes	49 (11.1)	26 (57.8)	19 (42.2)		30 (66.7)	15 (33.3)	
**ID Thief**							
No	440 (99.3)	282 (66.4)	143(34.6)	**0.048**	329 (77.4)	96 (22.6)	0.445
Yes	3 (0.7)	0 (0.0)	2 (100.0)		2 (100.0)	0 (0.0)	
**Never**							
No	60 (13.5)	31 (56.4)	24 (43.6)	0.104	37 (67.3)	18 (32.7)	0.051
Yes	383 (86.5)	251 (67.5)	121(32.5)		294 (79.0)	78 (21.0)	
**Over the last 2 weeks**							
No	49 (84.5)	26 (59.1)	18 (40.9)	0.419	29 (65.9)	15 (34.1)	0.487
Yes	9 (15.5)	4 (44.4)	5 (55.6)		7 (77.8)	2 (22.2)	
**Bystanders of Harassment**							
No	333 (74.7	221 (68.4)	102(31.6)	0.061	257 (79.6)	66 (20.4)	0.092
Yes	113 (25.3)	62 (58.5)	44 (41.5)		76 (71.7)	30 (28.3)	
**Defamation**							
No	230 (51.6)	152 (68.2)	71 (31.8)	0.318	176 (78.9)	47 (21.1)	0.501
Yes	216 (48.4)	131 (63.6)	75 (36.4)		157 (76.2)	49 (23.8)	
**Non-consensual sharing of intimate content**							
No	369 (82.7)	247 (69.0)	111(31.0)	**0.003**	287 (80.2)	71 (19.8)	**0.005**
Yes	77 (17.3)	36 (50.7)	35 (49.3)		46 (64.8)	25 (35.2)	
**Stalking**							
No	372 (83.4)	247 (69.0)	111(31.0)	**0.003**	289 (80.7)	69 (19.3)	**0.001**
Yes	74 (16.6)	36 (50.7)	35 (49.3)		44 (62.0)	27 (38.0)	
**Discrimination**							
No	161 (36.1)	111 (71.2)	45 (28.8)	0.087	128 (82.1)	28 (17.9)	0.096
Yes	285 (63.9)	172 (63.0)	101(37.0)		205 (75.1)	68 (24.9)	
**Insult**							
No	161 (36.1)	114 (73.5)	41 (26.5)	**0.013**	126 (81.3)	29 (18.7)	0.170
Yes	285 (63.9)	169 (61.7)	105 (38.3)		207 (75.5)	67 (24.5)	
**ID Thief**							
No	307 (68.8)	212 (71.4)	85 (28.6)	**< 0.001**	231 (77.8)	66 (22.2)	0.908
Yes	139 (31.2)	71 (53.8)	61 (46.2)		102 (77.3)	30 (22.7)	
**Never**							
No	344 (77.1)	209 (63.7)	119(36.3)	0.077	253 (77.1)	75 (22.9)	0.662
Yes	102 (22.9)	74 (73.3)	27 (26.7)		80 (79.2)	21 (20.8)	
**Over the last 2 weeks**							
No	178 (52.5)	112 (65.1)	60 (34.9)	0.657	141 (82.0)	31 (18.0)	**0.042**
Yes	161 (47.5)	96 (62.7)	57 (37.3)		111 (72.5)	42 (27.5)	

P-value obtained via chi-squared test.
Overall sample: column percentage
Risk of anxiety (GAD-2)/ Risk of depression (PHQ-2): row percentage
Abbreviation **GAD-2** Generalized Anxiety Disorder 2-item; **PHQ-2** Patient Health Questionnaire 2-item; **ID** identity

Supplementary Table S7. Risk of anxiety and depressive disorders (GAD-2 and PHQ-2 score) and face-to-face aggression and access to mental health service.

**Characteristic**	**Overall sample**	**Risk of anxiety (GAD-2)**			**Risk of depression (PHQ-2)**		
		**No**	**Yes**	**p-value**	**No**	**Yes**	**p-value**
	**N (%)**	**N (%)**	**N (%)**		**N (%)**	**N (%)**	
	N=446	283 (66.0)	146 (34.0)		333 (77.6)	96 (22.4)	
**FtF violence Victims**							
Never	207 (46.9)	143 (70.8)	59 (29.2)	0.087	161 (79.7)	41 (20.3)	0.651
Only childhood	184 (41.7)	112 (62.6)	67 (37.4)		137 (76.5)	42 (23.5)	
Only Adulthood	19 (4.3)	12 (70.6)	5 (29.4)		13 (76.5)	4 (23.5)	
Both	31 (7.0)	15 (50.0)	15 (50.0)		21 (70.0)	9 (30.0)	
**FtF violence Authors**							
Never	385 (87.1)	249 (66.6)	125 (33.4)	0.329	290 (77,5)	84 (22,5)	0,753
Only childhood	49 (11.1)	28 (59.6)	19 (40.4)		36 (76,6)	11 (23,4)	
Only Adulthood	4 (0.9)	2 (50.0)	2 (50.0)		3 (75,0)	1 (25,0)	
Both	4 (0.9)	0 (0.0)	4 (100.0)		4 (100,0)	0 (0,0)	
**FtF violence Bystanders**							
Never	141 (32.0)	105 (77.8)	30 (22.2)	**< 0.001**	115 (85,2)	20 (14,8)	**0,033**
Only childhood	189 (43.0)	114 (62.0)	70 (38.0)		139 (75,5)	45 (24,5)	
Only Adulthood	49 (11.1)	34 (72.3)	13 (27.7)		36 (76,6)	11 (23,4)	
Both	61 (13.9)	28 (45.9)	33 (54.1)		41 (67,2)	20 (32,8)	
**Context in which generally CyA occurs according to respondents' opinion**							
Online	230 (52.0)	154 (68.1)	72 (31.9)	0.593	177 (78,3)	49 (21,7)	0,928
In person	81 (18.3)	47 (62.7)	28 (37.3)		58 (77,3)	17 (22,7)	
Unknown	131 (29.6)	82 (64.1)	46 (35.9)		98 (76,6)	30 (23,4)	
**Access to Mental Health Service**							
No	340 (80.8)	240 (70.6)	100 (29.4)	**<0.001**	272 (80.0)	68 (20.0)	**0.010**
Yes	81 (19.2)	36 (44.4)	45 (55.6)		54 (66.7)	27 (33.3)	
**PHQ-2**							
Negative	333 (77.6)	264 (79.3)	69 (20.7)	**<0.001**	-	-	-
Positive	96 (22.4)	19 (19.8)	77 (80.2)				

P-value obtained via chi-squared test.
Overall sample: column percentage
Risk of anxiety (GAD-2)/ Risk of depression (PHQ-2): row percentage
Abbreviation **GAD-2** Generalized Anxiety Disorder 2-item; **PHQ-2** Patient Health Questionnaire 2-item; **FtF** face to face; **CyA** cyber aggression
